# Cost-effectiveness of a programme to address sedentary behaviour in older adults: results from the SITLESS RCT

**DOI:** 10.1093/eurpub/ckac017

**Published:** 2022-04-15

**Authors:** Manuela Deidda, Laura Coll-Planas, Mark A Tully, Maria Giné-Garriga, Frank Kee, Marta Roqué i Figuls, Nicole E Blackburn, Míriam Guerra-Balic, Dietrich Rothenbacher, Dhayana Dallmeier, Paolo Caserotti, Mathias Skjødt, Emma McIntosh, the SITLESS Consortium

**Affiliations:** Health Economics and Health Technology Assessment (HEHTA), Institute of Health and Wellbeing (IHW), University of Glasgow, Glasgow, UK; Fundació Salut i Envelliment—UAB, Institute of Biomedical Research (IIB Sant Pau), Universitat Autònoma de Barcelona, Barcelona, Spain; University of Ulster, Coleraine, Londonderry, UK; Facultat de Psicologia, Ciències de l’Educació i de l’Esport Blanquerna (Universitat Ramon Llull), Barcelona, Spain; UKCRC Centre of Excellence for Public Health (NI), Centre for Public Health, School of Medicine, Dentistry and Biomedical Sciences, Queen's University Belfast, Belfast, UK; Fundació Salut i Envelliment—UAB, Institute of Biomedical Research (IIB Sant Pau), Universitat Autònoma de Barcelona, Barcelona, Spain; University of Ulster, Coleraine, Londonderry, UK; Facultat de Psicologia, Ciències de l’Educació i de l’Esport Blanquerna (Universitat Ramon Llull), Barcelona, Spain; Institute of Epidemiology and Medical Biometry, Ulm University, Ulm, Germany; Ulm University, Ulm, Baden-Württemberg, Germany; Agaplesion Bethesda Hospital, Geriatric Research Unit Ulm University, Ulm, Germany; Department of Sports Science and Clinical Biomechanics, Syddansk Universitet, Odense M, Denmark; Department of Sports Science and Clinical Biomechanics, Syddansk Universitet, Odense M, Denmark; Health Economics and Health Technology Assessment (HEHTA), Institute of Health and Wellbeing (IHW), University of Glasgow, Glasgow, UK

## Abstract

**Background:**

This study details the within-trial economic evaluation and long-term economic model of SITLESS, a multi-country, three-armed randomized controlled trial comparing a combined intervention of exercise referral schemes (ERS) enhanced by self-management strategies (SMS) against ERS alone and usual care (UC).

**Methods:**

A cost-utility analysis, conducted from the base-case perspective of the National Health Service and personal and social services, estimated the incremental cost per incremental quality-adjusted life year (QALY) and years in full capability (YFC). A secondary analysis combined the costs with a broad set of outcomes within a cost-consequence framework, from a societal perspective. A Markov-type decision-analytic model was developed to project short-term changes in physical activity to long-term outcomes and costs, over a 5- and 15-year time horizon.

**Results:**

The results of the within-trial analysis show that SMS+ERS is highly likely to be cost-effective compared to ERS alone (ICER €4270/QALY), but not compared to UC. Participants allocated to the SMS+ERS group also showed an improvement in YFC compared to ERS alone and UC. The long-term analysis revealed that SMS+ERS is likely to be a cost-effective option compared to ERS and UC over a 5-year, but not with a 15-year horizon, being then dominated by ERS alone.

**Conclusion:**

This research provides new evidence that SMS is a cost-effective add-on to ERS strategies. This economic evaluation informs the case for further, cost-effective, refinement of lifestyle change programmes targeted to older adults, with the aim of ultimately reducing the impact of non-communicable diseases in this population.

## Introduction

Being insufficiently physically active is a known risk factor for major chronic diseases, disability and death, causing 9% of premature mortality worldwide.^1^ In Europe, an inactive lifestyle resulted in a cost of 80.4 billion Euro in 2012.^2^ In addition to low physical activity (PA), sedentary behaviour (SB), may also pose a significant health risk, independent of meeting the recommended levels of PA^3^ The cost impacts of prolonged SB to health services are also considerable, reaching £0.7 billion (∼0.8 billion Euro^4^).

Given the detrimental health and economic consequences of insufficient PA and high levels of SB, the evaluation and economic evaluation of interventions promoting active lifestyles is a key public policy research priority. This is increasingly relevant for the older population, who represent the fastest growing segment of the world population and account for almost 40% of the total healthcare expenditure across Europe.^5^

An active lifestyle has been identified as a key modifiable factor to attenuate decline in physical and mental health in older adults^6^ leading to healthy ageing trajectories^7^ by increasing the odd of improving health and functioning. A recent systematic review^8^ investigated the association between PA and healthy ageing, showing that adults engaged in high levels of PA have a 39% higher probability of living a healthy life than their inactive counterparts.

Indeed, increased PA and reduced SB in older adults prevent cognitive and functional decline, alleviate the symptoms of various chronic conditions associated to old age and might prevent or even reverse frailty.^9^ This ultimately leads to improvements in the quality of life and wellbeing of older adults and an ‘active and healthy ageing’.^10^

In the last 20 years, exercise referral schemes (ERS) have become widely implemented in Europe as a public health programme to encourage an active lifestyle.^11^ Within an ERS programme, individuals assessed as insufficiently active are referred by primary care providers to PA programmes provided by a leisure facility or another third-party service.^12^ Although ERS have been demonstrated to be potentially effective and cost-effective in the older adult population over the short term,^13–16^ lack of commitment over the long term has been identified as a major barrier to the effectiveness and cost-effectiveness of ERS. In this regard, behavioural interventions in the form of self-management strategies (SMS) are aimed to increase motivation, thus promoting sustained behaviour change over time.^12^^,^^17^ SMS strategies have been found to be effective to reduce SB in adults^18^ and in the older adults population.^19^ However, evidence on the cost-effectiveness of SMS intervention in community-dwelling healthy older adults is scarce in the healthy ageing literature, as it focuses on the general adult population or those with chronic conditions.^15^^,^^20^

The SITLESS randomized controlled trial (RCT) aimed to determine whether and at what cost ERS can be enhanced by SMSs to reduce SB, increase PA and improve markers of health, quality of life and function in community-dwelling older adults from four European countries, comparing a combined intervention of SMS + ERS against ERS alone and general recommendations about PA [usual care (UC)].

This article reports the economic evaluation conducted alongside the SITLESS multinational RCT and includes a within-trial economic analysis, evaluating the cost-effectiveness of SMS + ERS vs. ERS and UC using an intention-to-treat analysis and a long-term model extrapolating the cost-effectiveness results beyond the within-trial component. A health economics protocol, describing the planned health economics analysis, has been published.^21^

This appears to be one of the first economic evaluations assessing the value for money generated by a behavioural intervention targeting the older population in a multi-country European setting, thus generating increased understanding on the potential costs, cost savings and broader health and wellbeing outcomes generated by such intervention.

## Methods

### The SITLESS trial

SITLESS is a multi-country RCT with nested economic evaluation conducted in four centres located in four European countries: Belfast (UK), Barcelona (Spain), Ulm (Germany) and Odense (Denmark). Community-dwelling adults aged 65+, insufficiently active and/or self-reporting spending more than 6 h/day in SB, and without major physical limitations were randomized into three groups: SMS + ERS, ERS and UC.

The SMS + ERS participants received, concurrently to the 16-week PA programme offered to ERS participants, a 30-week SMS intervention encompassing a face-to-face visit, six-group sessions and four telephone calls. Participants allocated to the UC control group were offered two health advice meetings with general recommendations on healthy lifestyle. Participants’ assessments have been conducted at baseline, 4 months post-intervention, month 16 (12 months post-intervention) and month 22 (18 months post-intervention). Full details of the RCT protocol are reported elsewhere.^22^

### Within-trial analysis

#### Economic evaluation frameworks

The main economic evaluation framework was a CUA, combining costs to the National Health Service (NHS) and social care with Quality Adjusted Life Years (QALYs) and ‘Years of full capability’ (YFC).^23^ Results were reported in terms of the incremental cost per additional QALY/YFC generated by the intervention. A secondary analysis combined the costs and cost saving generated by the intervention with a broad set of effectiveness outcomes (health related, behavioural, functional) within a CCA framework.^24^^,^^25^

Using NICE guidance for the economic evaluation of public health interventions,^25^ the perspective of the NHS and Personal Social Services was used in the base-case analysis, including costs related to the usage of health care resources (general practitioner, nurse, social worker, physiotherapist, occupational therapist, day hospital, psychiatrist, hospital outpatient clinic, A&E) and usage of social services (home care/home help, home meal delivery, day centre, meals provided at community centres, night care). A sensitivity analysis considering a broader societal perspective (i.e. accounting for care expenses sustained by individuals, besides those sustained by the NHS and social services^25^) was also conducted, adding the personal costs of attending exercise facilities, opportunity cost of exercise, additional costs associated with increasing PA/reducing SB and the cost of informal care.

#### Identifying, measuring and valuing costs and outcomes

##### Outcomes

The outcomes included in the CUA analysis were QALYs and YFC,^26^ calculated from EuroQol (EQ-5D-5L)^27^ and the ICEpop CAPability measure for Older people (ICECAP-O).^26^ EQ-5D-5L and ICECAP-O scores were calculated at each time point and combined with time, using the area under the curve approach, to generate QALYs and YFC.^23^^,^^28^ Utility scores were calculated by mapping the 5L descriptive system data onto the 3L value set, using the country-specific ‘crosswalk’ value sets.^29^ Given the multinational aspect of the analysis, in the base-case analysis, EQ-5D utility scores were derived using country-specific EQ-5D tariffs, which reflect country-specific differences in health perceptions and preferences. In the absence of country-specific utility weights to value ICECAP-O, UK value sets have been used for consistency across all four countries.

##### Costs

The cost of the SITLESS intervention and the comparators (ERS; UC) was collected using tailored cost logs, which captured the relevant centre-specific cost components, including actual costs (e.g. cost of the venue, number and type of staff involved in delivering the intervention, contact duration, travel costs sustained by participants and staff, average cost of the equipment used), as well as the opportunity costs (i.e. the foregone benefit of option not chosen), which was included in place of the actual cost in a deterministic sensitivity analysis. Specifically, the opportunity costs associated with the intervention costs (e.g. venue, travel time of staff) as well as the opportunity costs associated with the use of private resources (e.g. caregivers time; participants travel time) have been estimated. The opportunity cost of exercise reflects the value of time lost by participants participating in the SITLESS intervention, who could have used their time in a different way (working, doing other leisure time activities, etc.). An average hourly wage rate was used to estimate this opportunity cost of time. The opportunity cost of the venue reflects the value of alternative usage of the venue (e.g. community activities, breastfeeding courses) to reflect the true cost of providing the SITLESS venue costs when rolled out.

Individual-level resource-use data were collected prospectively within trial at all relevant time points, using a data collection instrument tailored to capture country-specific differences (e.g. inclusion of country-specific examples of social services) as well as usage of health and community services, use of exercise facilities, opportunity cost of exercise, additional costs sustained to increase PA/reduce SB.

Following recommended practices to evaluate resources in multinational RCTs,^30^ a multi-country costing approach was adopted, using country-specific unit cost estimates to evaluate the resources used in the SITLESS countries. All price weights were converted into a common currency (Euro) by use of Purchasing Power Parity (PPP) statistics reported by the OECD for a base year (2017). The OECD consumer price index for each country^31^ was used to inflate unit costs estimates retrieved from previous studies.

#### Economic evaluation analysis methods

Incremental mean QALYs, YFC and costs between treatment groups were estimated on a multiple imputed dataset, adjusting for baseline utility and baseline covariates, using a multi-level GLM model (MGLM) to account for the complex hierarchical nature of the data as well as non-normality of outcomes.

A detailed description of statistical methods, including missing data imputation, has been included in Supplementary appendix S1.

The incremental cost-effectiveness ratio (ICER) was calculated for the two comparison groups, providing an estimate of the additional cost per additional QALY (or YFC) generated by the SMS intervention. ICERs were plotted in the cost-effectiveness plane and compared with country-specific thresholds. Being an explicit Willingness to pay (WTP) threshold not available for all countries except UK, country-specific GDP/capita levels have been used,^32^ and cost-effectiveness is assessed by considering the highest and lowest thresholds. The cost/YFC ICER was compared against the £33 500–£36 150 threshold range estimated by Kinghorn and colleagues.^33^

### Long-term model

The long-term analysis explores the likely long-term cost-effectiveness of SMS + ERS beyond the 22 months’ time horizon by extrapolating short-term changes in PA and SB into longer-term outcomes (i.e. mortality, quality-adjusted life expectancy) and costs, considering a 5- and 15-year time horizon.

A full description of the SITLESS Markov model has been included in Supplementary appendix S2.

### Sensitivity analysis

Probabilistic sensitivity analysis (PSA) was performed to quantify the joint effect of uncertainty around costs and QALYs.^34^ Bootstrapping was used to generate 1000 cost-QALY pairs, which were then represented graphically in a cost-effectiveness plane and translated into cost-effectiveness acceptability curves (CEACs), indicating the probability that each intervention is cost-effective for a range of cost-effectiveness threshold values.

The deterministic sensitivity analysis explores several scenarios in, including inclusion of a broader range of costs (societal perspective), inclusion of falls-related medical costs only, 20% variation around the cost of the SMS intervention, inclusion of the opportunity cost of the intervention (included as a ‘proxy’ cost, estimating the benefit foregone by using the resources to deliver the SITLESS intervention, as opposed to the actual cost), sensitivity of results to departures from the missing not at random hypothesis.

## Results

### Within trial

The average cost per person of the SMS and UC intervention is similar across countries, ranging from €121.9–141.3 (SMS) to €10.3–20 (UC). The ERS intervention shows a larger range (€112.3–239.4), with the lowest value seen in Spain (€112.3) mainly due to the absence of participants travel costs (all participants could walk to the sessions) and lower venue rental costs. The staff costs involved with delivering the intervention represent the main cost component, accounting for more than 50% of all country total costs. Tables 1, 2 and 3 in Supplementary appendix S3 show a detailed breakdown of the cost of the SMS+ERS, ERS and UC intervention, respectively, for each country.

**Table 1:** Incremental cost and outcome (QALY, YFC) summary, SMS+ERS vs. UC; SMS+ERS vs. ERS

Results table: within trial cost, QALY, YFC and ICER (SMS+ERS vs. UC)															
COST/QALY															
	Arm						Probability SMS+ERS cost effective								
	ERS+SMS	UC	Difference	95% bootstrapped CI		ICER	WTP € 20 433			WTP € 30 650	WTP € 27 300		WTP € 37 700		
**Cost**	3171	2672	**499**	-56	1143	**37519**	**22%**			**35%**	**31%**		**42%**		
**QALY**	1.449	1.4357	**0.0133**	-0.015	0.036										

**COST/YFC**															

** **							**Probability SMS+ERS cost effective**								
	**ERS+SMS**	**UC**	**Difference**	**95% bootstrapped CI**		**ICER**	**WTP £34 255**				**WTP £36 932**				

**Cost**	3171	2672	**499**	-56	1143	**108478**	**39%**				**41%**				
**YFC**	1.4957	1.4911	**0.0046**	-0.013	0.027										

**Results table: within trial cost, QALY, YFC and ICER (SMS+ERS vs. ERS)**															

	**Arm**		** **				**Probability SMS+ERS cost effective**								
	**ERS+SMS**	**ERS**	**Difference**	**95% bootstrapped CI**		**ICER**	**WTP € 20 433**			**WTP € 30 650**	**WTP € 27 300**		**WTP € 37 700**		

**Cost**	3171	3057	**114**	-424	626	**4270**	**86%**			**91%**	**89%**		**92%**		
**QALY**	1.449	1.4223	**0.0267**	-0.001	0.055										

**COST/YFC**															

** **								**Probability SMS+ERS cost effective**							
	**ERS+SMS**	**ERS**	**Difference**	**95% bootstrapped CI**		**ICER**	**WTP £34 255**				**WTP £36 932**				

**Cost**	3171	3057	**114**	-424	626	**8571**	**91%**				**92%**				
**YFC**	1.4957	1.4824	**0.0133**	-0.004	0.036										

**Table 2:** Cost-consequence balance sheet, 18-month follow-up

	SMS+ERS vs. ERS	Improvement	SMS+ERS vs. UC	Improvement
Incremental costs				
Healthcare perspective	€−97.4 (378.9)	Yes	€−90.1 (372.02)	Yes
Societal perspective	€−526 (864.9)	Yes	€−609.5 (729.9)	Yes
Intervention cost	€100.16 (3.09)	No	€296.4 (1.98)	No
Incremental consequences				
% Sedentary time	−0.457 (0.739)	Yes	−0.817 (0.761)	Yes
% Moderate-vigorous activity	0.109 (0.262)	Yes	0.177 (0.242)	Yes
% Light activity	0.349 (0.588)	Yes	0.642 (0.635)	Yes
Time sedentary (hours/week, self-reported)	0.142 (0.231)	No	−0.328^a^ (0.253)	Yes
Anxiety score (HADS)	−0.235 (0.374)	Yes	−0.172 (0.398)	Yes
Depression score (HADS)	−0.019 (0.335)	Yes	−0.255 (0.359)	Yes
Activities of daily living (ADL) score	−0.304 (0.369)	Yes	0.161 (0.324)	No
Falls efficacy scale (FESI) score	0.013 (0.381)	No	−0.066 (0.385)	Yes
Social network score (Lubben)	0.515 (0.642)	Yes	0.317 (0.778)	Yes

SBQ: sedentary behaviour questionnaire, self-reported hours/day of SB; scale 0–24.

HADS: anxiety and depression score; scale: 0 (no anxiety) to 21.

FESI: short falls efficacy; scale: 7 (no fear of falling) to 28.

ADL: activities of daily living; scale 0 (independence) to 24.

Lubben social network scale: measures social isolation; scale: 0 (greater social isolation) to 30.

Standard errors reported in parentheses.

a Significance at 10% level.

**Table 3:** Incremental costs, incremental QALY and ICER for the base-case scenario

	Arm	Total Cost (€)	Total QALY	Comparison	Incremental Cost (CI)	Incremental QALY (CI)	ICER
5 YEARS Time horizon	SMS+ERS	13,294	2.6584				
Base-case analysis	ERS	13,326	2.6549	SMS+ERS vs ERS	-32 (-140; 61)	0.0035 (-0.0072; 0.0150)	SMS+ERS dominates
	UC	13,347	2.6526	SMS+ERS vs. UC	-52 (-180; 51)	0.0058 (-0.0045; 0.0189)	SMS+ERS dominates
15 YEARS Time horizon	SMS+ERS	45,634	6.9570				
Base-case analysis	ERS	45,604	6.9590	SMS+ERS vs. ERS	30 (-186; 249)	-0.0020 (-0.0355; 0.0330)	SMS+ERS is dominated
	UC	45,628	6.9538	SMS+ERS vs. UC	6 (-236; 255)	0.0032 (-0.0336; 0.0405)	1960

Credibility interval in parenthesis.

### Cost-utility analysis

Table 1 outlines the incremental costs and outcome (QALY and YFC) and the ICER for the comparisons SMS + ERS vs. UC and SMS + ERS vs. ERS.

As shown in table 1, when controlling for baseline covariates, the participants randomized to SMS + ERS accrued greater incremental costs (€499) and also reported greater quality of life than participants randomized to UC. The PSA (Supplementary appendix S7) reveals the majority of cost-effectiveness pairs lying in the north-east quadrant, showing little uncertainty regarding the improvement in quality of life associated with the SMS + ERS intervention. The ICER (€37 519/QALY) is above all the country-specific WTP threshold except the highest boundary. This is also reflected in the CEACs, where the probability of SMS + ERS being cost-effective compared to UC is below 42%.

The participants randomized to SMS + ERS accrued incremental costs of €114 and an incremental quality of life of 0.0267 compared to participants randomized to ERS. The cost-effectiveness plane reveals the majority of cost-effectiveness pairs lying in the north-east quadrant, showing little uncertainty regarding the improvement in quality of life associated with the SMS + ERS intervention combination. For the SMS + ERS vs. ERS comparison, the ICER (€4270/QALY) is below all the conventional cost-effectiveness thresholds. Considering the CEAC for this comparison (figure 1), SMS + ERS is the optimal intervention combination, with a likelihood of being cost-effective above 85% for all the WTP threshold values. Table 1 also shows that participants in the SMS + ERS group reported a higher level of capability wellbeing (although not statistically significant) than participants randomized to ERS and UC. As shown in Supplementary appendix S4, the cost-effectiveness results obtained in the base-case scenario are robust to several scenario analyses.

Considering the results for YFC, the estimated ICER for the SMS + ERS vs. UC comparison is €108478/QALY, which is above the YFC threshold range,^33^ whereas the ICER for the SMS + ERS vs. ERS comparison is €8571, which is below the YFC threshold range, making this option highly cost-effective. The likelihood of SMS + ERS being cost-effective ranges between 39% and 41% in the comparison with UC and between 91% and 92% in the comparison with ERS (table 1).

### CCA

Table 2 shows the cost-consequence balance sheet, i.e. mean difference between arms at 22-month follow-up, in terms of costs and a broad set of efficacy outcomes.

At 18 months post-intervention, participants allocated to the SMS + ERS arm showed on average lower, although not statistically significant, healthcare and societal costs than participants allocated to ERS and UC. Also, the SMS + ERS arm shows an improvement, albeit not statistically significant in most cases, in most of the outcomes, including an increase in the amount of time spent doing light, moderate and vigorous intensity PA; reduction of anxiety and depression; increase in independence; and improvement of social network. A reduction in SB is only observed when SB is objectively assessed with accelerometers. When self-reported SB is considered, a statistically significant reduction in the average number of hours spent doing sedentary activities is only observed in the comparison SMS + ERS vs. UC. A reduction in the fear of falling is also observed, but only in the comparison with UC arm.

### Long-term model

Table 3 shows the long-term cost-effectiveness results for the base-case analyses. In the 5-year base-case scenario, total costs for the SMS + ERS group were €13 294 as compared to €13 326 in the ERS group and €13 347 in the UC group. SMS + ERS generates very small cost savings, compared to ERS (€32) and UC (€52). Total QALYs in the SMS + ERS group were 2.658, as compared to 2.6549 in the ERS group and 2.6526 in the UC group, resulting in a small gain of 0.0035 QALY per participant in the comparison SMS + ERS vs. ERS and 0.0058 in the comparison SMS + ERS vs. UC. Being less costly and more effective, SMS + ERS dominates ERS and UC. In the 15-year base-case scenario, the total cost for the SMS + ERS group was €45 634 as compared to €45604 (ERS) and €45628 (UC). SMS + ERS has an incremental cost of €30 compared to ERS and €6 compared with UC. Total QALY in the SMS + ERS group were 6.957, as compared with 6.959 in the ERS group and 6.9538 in the UC group. This results in a small utility decrement of 0.002 in the comparison SMS + ERS vs. ERS and a utility gain of 0.0032 in the comparison SMS + ERS vs. UC. When considering a 15-year time horizon, SMS + ERS is dominated by ERS, being less effective and more costly. However, when comparing SMS + ERS with UC the ICER is €1960/QALY, which is below the conventional WTP thresholds, hence reflecting a cost-effective option. Compared with the 5-year scenario, SMS + ERS no longer dominates the comparators. This is due to the fact that the initial comparative advantage of SMS + ERS in terms of PA levels improvement diminishes after the first year. These results are robust to several scenario analyses (Supplementary appendix S5).

## Discussion

The results of the short-term within-trial cost-effectiveness analysis reveal that adding the behavioural SMS component to the ERS intervention is cost-effective when compared with ERS alone. However, when comparing SMS + ERS vs. UC, SMS + ERS leads to small increases in quality of life and does not generate sufficient cost savings to be cost-effective. This result aligns with a previous trial^35^ conducted in a younger population that showed that advice and information on PA dominates exercise referral programmes in terms of cost-effectiveness, while they were equal in terms of effectiveness on health outcomes. Also, the modest improvements in quality of life and cost savings generated by the intervention are in line with previous studies suggesting that the cost-effectiveness of such preventive interventions on a relatively healthy population is subject to considerable uncertainty, being strongly dependent on small changes in cost or outcome measures.

The descriptive evidence provided by the CCA suggests that in the 22-month follow-up participants to the ERS + SMS intervention performed relatively better (albeit differences were not statistically significant) than those in the comparison groups across a preponderance of outcomes of interest including PA levels, SB, fear of falling, anxiety and depression, showing at the same time lower healthcare and social costs. Although in the absence of a formal rule to weight such ‘consequences’ the decision maker cannot formally appraise the results of the CCA (as for the CUA), the CCA provides complementary evidence on the broader benefits (beyond QALY and YFC) generated by SMS + ERS vs. comparisons.

The findings of the long-term cost-effectiveness analysis reveal that the SMS + ERS intervention combination is likely to be a cost-effective option compared to both ERS alone and UC in the medium term (5 years) but is dominated by ERS alone over a 15-year time horizon. Unlike other studies,^36^ the SITLESS model explicitly modelled decrease in the intervention effect over time, using transition rates across PA states calculated within-trial. This is a strength of our analyses, which has benefitted from a follow-up period greater than 1 year to model the decay of the intervention. Also, when comparing SMS + ERS to UC, SMS + ERS generates improvements in QALY and cost savings, but there is considerable uncertainty, reflected in a relatively low probability of cost-effectiveness, which prevent us to conclude that the intervention is cost-effective. Among the limitations of the long-term model cost-effectiveness analysis, the assumptions made in relation to the decay of the intervention effect over time have a strong impact on the cost-effectiveness results. Also, while base-case results seem to suggest evidence of cost-effectiveness of SMS + ERS vs. both comparators, the QALY improvements and costs savings are modest. Therefore, even in the most favourable scenario, such small changes in costs and QALY might not justify implementing a resource-intense intervention as the SMS + ERS intervention.

Borrowing insights from social cognitive theory, the SITLESS trial added a behavioural component, in the form of SMS, to the already existing ERS schemes, but heterogeneously implemented across European countries.

The economic evaluation alongside SITLESS is the first economic evaluation to assess the value added by a behavioural intervention to ERS, in a population of a community-dwelling older adults, as compared to ERS alone and UC, adopting a methodology suited to the economic evaluation of a complex public health intervention.^37^ This is the first multi-country study of its type, exploring a heterogeneous setting in terms of cultural values, attitudes towards physical exercise, healthcare and social settings and generating potentially generalizable results.

Overall, this research provides new evidence that SMS + ERS, compared to ERS alone, is likely to generate value for money in the short term, in terms of improvements in quality of life, wellbeing and capability. Also, SMS + ERS has the potential to be cost-effective compared to ERS alone and UC (which is a dominated intervention) in a medium-term time horizon (5 years), generating modest increases in QALY and cost savings, although benefits and cost savings are not likely to be sustained in the longer term, due to an expected reduction over time in the rate of participants moving to a higher PA level in the SMS + ERS group compared to ERS and UC.

It is worth noting that the cost-effectiveness results are based on commonly accepted NICE thresholds (£20 000–£30 000). Using a ‘supply-based’ threshold of £12 000^38^ the SITLESS intervention is less likely to be cost-effective.

Considering this, European decision-makers may consider the incremental cost per QALY worth the investment, supporting the enhancement of ERS interventions—often implemented as part of public health programmes—with an SMS behavioural component as a likely highly cost-effective strategy within a community-dwelling 65+ European population.

The evidence generated from the SITLESS trial provides new economic evidence for investing in interventions to increase PA and reduce SB thereby reducing the impact of non-communicable diseases in this population. This will allow the further refinement of lifestyle change programmes targeted to older adults, grounded on the behavioural theory.

## Supplementary data

Supplementary data are available at *EURPUB* online.

## Supplementary Material

ckac017_Supplementary_Data
