# Supplementary material for: Cost-effectiveness of a programme to address sedentary behaviour in older adults: results from the SITLESS RCT
Source: Eur J Public Health. 2022 Apr 15;32(3):415–21. doi: 10.1093/eurpub/ckac017 (PMC9159313; doi:10.1093/eurpub/ckac017)
Supplement: ckac017_Supplementary_Data [file ckac017_supplementary_data.docx]

**Supplementary Material**

**Supplementary Appendix S1: within-trial analysis: statistical and econometric methods**

**Economic evaluation analysis methods**

Outcomes and costs were analysed using multivariate, multilevel mixed-effects generalized linear model (MGLMs), adjusting for baseline characteristics. MGLMs allow the inclusion of both fixed effects and random effects, accounting at the same time for non-normality of costs and outcomes.

The choice of the statistical model to analyse cost and outcome data in SITLESS was driven by the existence of two levels of clustering. First, cluster at couple level: cohabiting individuals have been randomised together, implying a substantial correlation between subjects that needs to be considered. In addition, clustering at country level: the multicountry nature of the SITLESS intervention does require the between vs. within country correlation to be explicitly tackled. Cost and outcome data thus fall naturally in a complex hierarchical structure with three levels of nesting: individuals are nested within couples and couples are nested within countries. Using a multilevel model allows the correlation structure between participants and higher level units (couples and countries) to be explicitly modelled[1], tackling explicitly the lack of independence of errors between the observations.

Specifically, the outcomes and cost equation in the SITLESS analysis were analysed modelling countries as fixed effects and couples as random effects. A robustness analysis was performed including both couples and country as random effect, and including only country random effects, showing no significant differences in results. Also, the intra-class correlation between costs and outcomes in the same centre was very low, indicating that between-countries variation is not an important component of the total variation, i.e. countries do not differ substantially in measured outcomes and costs. The likelihood ratio test (which tests the null hypothesis of random effects being zero) reject the null hypothesis, thus providing evidence against the inclusion of random effects at country level. Following Briggs, Glick [2], a joint tests of significance for treatment-by-country interactions in the outcome and cost model was performed, to look for evidence of heterogeneity across countries for treatment effects in QALY or total costs, showing no significance, overall, of the treatment-centre interaction. This is in line with Drummond, Barbieri [3], who state that MLM may not be required when the number of countries in the trial is less than five. In this case, the level of within-country variability will be low relative to the between-country variability, providing evidence of low between-country heterogeneity. In this case, it’s likely that the pooled estimation would adequately represent the intervention effect across countries. Also, it is likely that the within-couples clustering effect plays a more important role in relation to costs, QALY and YFC than within-country heterogeneity.

The modified Park test was conducted to choose the best family, while a battery of test (Pearson correlation tests, Pregibon Link test and Modified Hosmer and Lemeshow test) were used to guide the choice of the best family. The Gamma family with log link was chosen for total costs and total YFC, while Poisson with log link was found to be the best fit for Total QALY, as these were the best fit according to the tests. Baseline costs, baseline EQ5D and ICECAP-O scores were included as covariate in the total cost, total QALY and total YFC regressions respectively, to account for potential imbalance in baseline utility, capability or resource use level [4]. All regressions were adjusted for baseline characteristics, including age, gender, marital status, education, a dummy indicating whether the individual lives alone and number of comorbidities.

All within-trial analyses were performed using Stata version 16.

**Missing data**

Following best practice [5] a thorough analysis has been performed in order to assess the extent of missingness, as well as the missing data mechanism. Total costs and total QALYs are cumulative quantities hence any missing data at any of the follow-up points results in those patient’s data being removed from the complete-case analysis. Further, since total QALY/YFC and total costs have been adjusted for baseline characteristics, those patients with missing values in any of the covariates which have been used in the regression have been dropped as well. Complete-case analysis consisted of the patients with completed data on baseline characteristics, who completed all EQ-5D and ICECAP-O profiles and have completed resource use data at each time point. Overall, there were 620 participants in the complete-case analysis (185 randomised to UC, 213 to ERS and 222 to SMS+ERS).

Table 1 below shows the percentage of missing data, by intervention arm, for baseline characteristics and total cost and outcomes (EQ5D and ICECAP-O scores), at each time point. The number of questionnaires returned at each follow-up point decreased with time: the percentage of missingness goes from less than 5% at baseline to almost 48% in the last follow-up. The table shows a consistent pattern across costs and outcomes, with a higher percentage of missing value in the UC arm. A proportion of missing data which differs by treatment allocation and across timepoints suggests that data are unlikely to be MCAR, making complete-case analysis biased. We further investigate the mechanism of missingness by assessing whether a significant association exist between: a) probability that total costs and outcomes are missing and baseline covariates; b) probability to observe missing observed outcomes and costs and previously observed outcomes and costs, finding that the probability of observing missing costs and outcomes is significantly associated with both baseline characteristics and, in most cases, with previously observed values of costs and outcomes, thus ruling out the possibility that data are MCAR and CD-MAR. The reasons discussed above, and, the large proportion of data lost for the complete-case analysis strengthen the rationale for using the multiple imputation data sets in the base case. While the analysis on the imputed dataset was performed as a base-case analysis, a sensitivity analysis considering a complete-case analysis was also performed.

Deterministic mean imputation was used to predict missing data at baseline[6]. Multiple imputation procedures using chained equations were used to impute follow-up missing data separately for each arm of the trial, creating 60 imputed datasets. Compared to other methods (e.g. mean imputation; last value carried forward), multiple imputation incorporates uncertainty associated with missing data, thus providing unbiased results[7]. Predictive mean matching has been used in order to deal with non-normality of cost and outcome data[5]. Schomaker and Heumann [8] approach (MI boot) procedure was used to calculate bootstrapped confidence intervals. This approach has been proved to yield valid inference when dealing with multiple imputed data.

Cost-effectiveness results obtained with the multiple imputation procedure strongly rely on the validity of the MAR assumption[5]: individuals who completed and returned all questionnaires are similar to the individuals who did not, conditional on their observed characteristics. However, this may not be the case: patients with missing quality of life, capability or cost data might be those with lower (or higher) quality of life or capability and accrued higher (or lower) healthcare and social costs.

Sensitivity analysis on the multiple imputation model has been thus performed to test how sensitive the cost-effectiveness results are to the MAR assumption, exploring sensitivity of results to departures from MAR [9]. Multiple imputation was performed in STATA 16, using the programme ‘mi impute chained’.

**Table 1: Missing data, by arm.**

| **Percentage of missing data, by arm** | | | |
| --- | --- | --- | --- |
|  | **UC** | **ERS** | **ERS+SMS** |
| **Outcomes** |  |  |  |
| EQ5D score -baseline | 5.46 | 3.58 | 4.18 |
| EQ5D score -post intervention | 34.06 | 20.13 | 23.08 |
| EQ5D score -12m | 49.13 | 37.36 | 37.58 |
| EQ5D score -18m | 47.82 | 39.82 | 39.12 |
| ICECAP-O score - baseline | 6.99 | 4.7 | 5.05 |
| ICECAP-O score - post intervention | 34.93 | 20.13 | 23.3 |
| ICECAP-O score -12m | 49.34 | 37.81 | 37.58 |
| ICECAP-O score - 18m | 47.82 | 40.04 | 39.56 |
|  |  |  |  |
| **Costs** |  |  |  |
| total cost1-baseline | 1.31 | 0.67 | 0.44 |
| total cost1-post intervention | 31.44 | 17.9 | 21.32 |
| total cost1-12m | 47.16 | 34 | 34.51 |
| total cost1-18m | 46.07 | 38.03 | 37.36 |
| total cost2-baseline | 1.31 | 0.67 | 0.44 |
| total cost2 -post intervention | 31.44 | 17.9 | 21.32 |
| total cost2-12m | 47.16 | 34 | 34.51 |
| total cost2 -18m | 46.07 | 38.03 | 37.36 |
| total cost3-baseline | 1.31 | 0.89 | 0.44 |
| total cost3-post intervention | 30.79 | 17.9 | 21.54 |
| total cost3-12m | 47.16 | 34 | 34.51 |
| total cost3-18m | 46.07 | 38.03 | 37.36 |
|  |  |  |  |
| **Baseline covariates** |  |  |  |
| age | 0.22 | 0 | 0 |
| male | 0 | 0 | 0 |
| marital | 5.68 | 2.46 | 3.3 |
| education | 2.18 | 2.01 | 1.32 |
| Living alone | 5.68 | 2.68 | 3.08 |
| comorbidity | 5.46 | 1.57 | 3.96 |

**Supplementary Appendix S2: The SITLESS long-term model**

1. **Model structure**

The SITLESS model is a Markov-type model projecting short-term changes in PA into longer terms outcomes (i.e. mortality, quality-adjusted life expectancy) and costs. The model simulated a cohort of community-dwelling adults aged 65+ years and includes eight mutually exclusive states (two physical activity states and six disease states) and death (an absorbing state).

The SITLESS long-term model has been adapted from existing models [10, 11] to account for the specificities of the SITLESS target population, including ageing-specific health outcomes and transition probabilities, informed by an ‘ad hoc’ systematic review.

Figure 1 shows the structure of the Markov model and Table 1 below shows the key assumptions underlying the model. The model has been developed using Microsoft Excel. We tested internal and face validity of the model

**Table 3: Model assumptions**

| Physical activity states | Physical activity has been classified into two health states using the metabolic equivalent (MET) as a unit of measurement that expresses the energy cost of physical activity.  In consideration of the physical activity recommendations for elderly adults [12], two physical activity states were included in the model: ‘active’ (>7.5 MET/hours/week, corresponding to 150 minutes of moderate intensity aerobic activity or 75 minutes of vigorous activity) and ‘inactive’ (<7.5 MET/hours/week). |
| --- | --- |
| Health states | The health states represent seven health conditions associated with a lack of physical activity in the older population: coronary heart diseases, stroke, type 2 diabetes, vascular dementia, Alzheimer`s disease, falls and fractures. The diseases to be included in the model were chosen – among the list of high-incidence diseases amongst the 65+ population - considering those for which an inactive lifestyle represented a key risk factor. Only final diseases and conditions (e.g. fractures, stroke), rather than intermediate states (e.g. osteoporosis, high blood pressure), were considered. The final set of diseases included in the model was co-created and validated by the SITLESS collaborators. |
| Transition between model states | At the beginning of the model, all members of the cohort were well and did not suffer from any of the diseases included in the model. Individuals start in a physical activity state, and at the end of the first cycle, participants can either stay in the same physical activity state, move to a different physical activity state, to a disease state or death. Once in a disease state, participants can either stay in the same disease state or move to the death state. While this assumption rules out the possibility that participant have more than one disease, comorbidities – which are common among the older adult population- have been taken into account in a sensitivity analysis, applying a utility decrement [80]. |
| Transitions between PA states | Calculated using the SITLESS RCT data;   - First year: probability of moving to a different PA state or staying in the same state considering the transitions between baseline and 4 months (post-intervention). - After the second year, as it is plausible to assume a decline in PA over time, we used the transitions between 4 months and 18 months follow-up to calculate transition probabilities. |
| Time horizon | 1. 5 years; b) 15 years. |
| Cycle length | 1 year |
| Time and cross-country adjustment | All costs included in the model are reported in Euros and adjusted using purchasing power parity (PPP) for a base year (2017). Costs and benefits are discounted at 1.5% per year [13]. |

Notes: One MET is defined as the ‘resting metabolic rate’ and is equivalent to consuming 3.5 mm of oxygen per kilogram of body weight.

**Figure 1 Markov model**


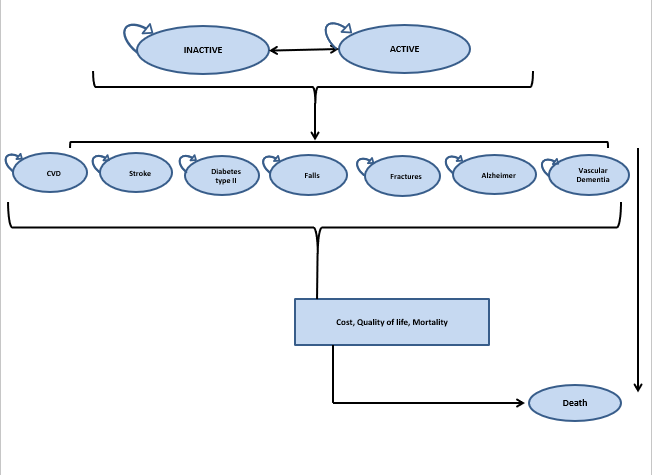


1. **Model parameters**

The parameters to populate the model have been retrieved from several sources, including the SITLESS RCT and ‘ad hoc’ systematic and scoping reviews.

Specifically:

- **Disease risk**

A systematic literature review was conducted to obtain an estimate of the risk of developing each of the chronic diseases identified as being relevant for the SITLESS 65+ target population by PA level.

A preliminary scoping review identified a set of chronic diseases associated with lack of physical exercise in older adults. The results of the review were validated through an expert opinion, in collaboration with the SITLESS team. The final set of diseases includes:

- Stroke
- Cardiovascular disease
- Alzheimer disease
- Vascular dementia
- Falls
- Fracture
- Diabetes type II

The search has been performed in 5 databases: Medline, Embase, Cochrane, Web of Science, Psychinfo and Sportdiscus. The search retrieved 23730 papers; after deleting 3790 duplicates, we end up with 19940 records.

The search was conducted in three phases:

1. Title screening: after excluding clearly irrelevant titles, 3052 abstracts were selected for abstract screening;

2. Abstract screening: we selected prospective studies discussing the impact of physical activity and sedentary behavior upon the risk of developing the previously identified diseases; 418 papers were selected for full text screening.

3. Full text screening:

a. we included only those studies not fulfilling the inclusion criteria. Specifically, we excluded papers: where the age at baseline was lower than 65, papers considering occupational activity only or where the impact of occupational activity could not be separated from leisure time activity; abstracts-only, conference proceedings or posters; cross-sectional studies.

b. We further excluded: papers where the risk of incident case of the disease was not specified; studies where physical activity was not assessed in METs (Metabolic equivalents), or in Kcal, times or intensity of physical exercise, which could be converted in MET; studies where the thresholds to identify physical activity levels were not stated (e.g. physical activity was classified as ‘high’ and ‘low’, without a quantification of PA states in terms of METs, duration or Kcal).

We finally included 12 papers discussing the differential risk of developing a disease based on physical activity levels including: 3 papers on CVD; 4 papers on Alzheimer; 3 papers on vascular dementia; 1 paper on falls; 1 paper on fracture.

No studies on diabetes and stroke fulfilled the inclusion criteria. Two studies considering a lower age threshold (60) have been thus retrieved from the literature search results. Among the included studies, only one study considered the impact of SB upon the risk of developing a disease [14].

Following the physical activity recommendations for older adults, two physical activities states were identified in the model: active, corresponding to at least 7.5 MET/hours/week, and inactive. The physical activity levels and corresponding incident cases reported in the studies have thus been aligned with the chosen ‘active’ and ‘inactive’ states. Specifically, for each category of PA reported in the study, a midpoint was calculated; incident cases were aggregated when necessary and allocated into the ‘active’ and ‘inactive’ group.

Physical activity levels were expressed in METs/hour/week using consistent conversions across all the studies. When physical activity levels were reported in terms of duration, frequency and intensity, these have been converted into METs using 6 METs as the score for vigorous or strenuous intensity activity and 3 METs for moderate intensity activity[15]. When physical activity was reported in terms of type of activity (e.g. walking, sport activity), these were mapped into MET level using standard classification codes[15]. In few instances[16, 17] physical activity was reported in terms of distance or time spent walking; in absence of information on intensity, a MET equal to 1.8 was used. When physical activity levels were reported in terms of Kcal, the UK national average of body weight was used for the conversion into METs.

A meta-analysis with random effect was carried out separately for each disease (CVD, Alzheimer, vascular dementia).

While the original scope of the review was to identify the joint effect of PA and SB (rather than their independent contribution) on the risk of developing chronic diseases, the evidence available for the 65+ population subgroup was limited: most of the retrieved papers estimated the disease risk given PA levels; only a few of them considered the impact of SB, whereas none of them considered their joint impact. In consideration of the limited evidence available for the older segment of the population, after applying the exclusion criteria, we included only papers considering PA. The complementary effect of SB has been taken into account in a scenario analysis, by applying a mortality correction [18].

- **Transitions between physical activity states**

Transitions between PA states, as well as the proportion of participants starting in the ‘Active’ and ‘Inactive’ states were calculated from the SITLESS RCT data. Although in the SITLESS trial, PA and SB have been assessed objectively (using Actigraph) and subjectively, the model employed the subjective measures of PA and SB for two main reasons: 1) hours of moderate and vigorous PA recorded by the accelerometer need to be adjusted by the wearing time, thus making it difficult comparisons across arms and time; 2) much of the evidence linking PA to disease risk uses self-reported measures, rather than objectively measured PA/SB [19].

- **Quality of life**

Utility values for the inactive and active states were calculated from the EQ-5D utility scores reported in the SITLESS RCT data.

To obtain the utility values associated with the chronic conditions considered in the model a scoping literature review was conducted. Amongst the studies identified by the review, systematic reviews were used in the first instance, if available (e.g. [20]); otherwise, parameters were retrieved from studies where the target population was close to the age of the SITLESS participants. Only when utility values for the 65+ population were not available, general adult population parameters were used.

- **Costs**

The costs associated with health states included in the model were retrieved from the literature, considering cost estimates including both direct medical costs and broader societal costs.

- Mortality

Mortality risk was estimated as an adjusted risk based on all-cause mortality combined with the age structure for the population[21], whereas cause-specific mortality risk was based on literature estimates (Table 2)

Table 2 summarises the parameters which have been used in the Markov model.

**Table 2 Markov model input parameters**

| **Model Parameters** | **Value** | **Distribution** | **95% CI** | | **SD** | **Source** |
| --- | --- | --- | --- | --- | --- | --- |
|  |  |  | Lower | Upper |  |  |
| **Transition probabilities (First year)** |  |  |  |  |  |  |
| **Physical activity states** |  |  |  |  |  |  |
| **SMS+ERS** |  |  |  |  |  |  |
| Inactive to inactive |  |  |  |  |  |  |
| Inactive to recommended activity | **0.54** | Lognormal | 0.44 | 0.64 |  | SITLESS RCT |
| Recommended activity to inactive | **0.25** | Lognormal | 0.18 | 0.32 |  | SITLESS RCT [22] |
| **ERS** |  |  |  |  |  |  |
| Inactive to recommended activity | **0.48** | Lognormal | 0.38 | 0.57 |  | SITLESS RCT [22] |
| Recommended activity to inactive | **0.35** | Lognormal | 0.27 | 0.44 |  | SITLESS RCT [22] |
| **UC** |  |  |  |  |  |  |
| Inactive to recommended activity | **0.41** | Lognormal | 0.27 | 0.56 |  | SITLESS RCT [22] |
| Recommended activity to inactive | **0.40** | Lognormal | 0.29 | 0.50 |  | SITLESS RCT [22] |
|  |  |  |  |  |  |  |
| **Transition probabilities (Year 2 and following)** |  |  |  |  |  |  |
| **Physical activity states** |  |  |  |  |  |  |
| **SMS+ERS** |  |  |  |  |  |  |
| Inactive to recommended activity | **0.29** | Lognormal | 0.22 | 0.37 |  | SITLESS RCT [22] |
| Recommended activity to inactive | **0.16** | Lognormal | 0.12 | 0.21 |  | SITLESS RCT [22] |
| **ERS** |  |  |  |  |  |  |
| Inactive to recommended activity | **0.40** | Lognormal | 0.32 | 0.48 |  | SITLESS RCT [22] |
| Recommended activity to inactive | **0.18** | Lognormal | 0.13 | 0.23 |  | SITLESS RCT [22] |
| **UC** |  |  |  |  |  |  |
| Inactive to recommended activity | **0.30** | Lognormal | 0.23 | 0.38 |  | SITLESS RCT [22] |
| Recommended activity to inactive | **0.14** | Lognormal | 0.10 | 0.20 |  | SITLESS RCT [22] |
|  |  |  |  |  |  |  |
| **Transition probabilities** |  |  |  |  |  |  |
| **Health States** |  |  |  |  |  |  |
|  |  |  |  | |  |  |
|  |  |  |  |  |  |  |
| Inactive to CVD | **0.0410** | Lognormal | 0.0279 | 0.0564 |  | meta-analysis[14, 23, 24] |
| Recommended activity to CVD | **0.0255** | Lognormal | 0.0172 | 0.0353 |  |  |
| Inactive to Alzheimer | **0.0110** | Lognormal | 0.0088 | 0.0135 |  | meta-analysis[16, 25, 26] |
| Recommended activity to Alzheimer | **0.0084** | Lognormal | 0.0059 | 0.0111 |  |  |
| Inactive to Dementia | **0.0057** | Lognormal | 0.0013 | 0.0129 |  | meta-analysis[16, 25, 27] |
| Recommended activity to Dementia | **0.0041** | Lognormal | 0.0004 | 0.0114 |  |  |
| Inactive to Falls | **0.1085** | Lognormal | 0.1039 | 0.1131 |  | Buchner, Rillamas‐Sun [28] |
| Recommended activity to Falls | **0.0776** | Lognormal | 0.0753 | 0.0799 |  |  |
| Inactive to Fractures | **0.0019** | Lognormal | 0.0012 | 0.0026 |  | Buchner, Rillamas‐Sun [28] |
| Recommended activity to Fractures | **0.0017** | Lognormal | 0.0013 | 0.0021 |  |  |
| Inactive to Stroke | **0.0069** | Lognormal | 0.0056 | 0.0083 |  | Jefferis, Whincup [29] |
| Recommended activity to Stroke | **0.0066** | Lognormal | 0.0052 | 0.0080 |  |  |
| Recommended activity to Diabetes | **0.0049** | Lognormal | 0.0047 | 0.0050 |  | InterAct Consortium [30] |
| Inactive to Diabetes | **0.0034** | Lognormal | 0.0033 | 0.0034 |  |  |
|  |  |  |  |  |  |  |
| **Costs (Annual cost, 2017, €)** |  |  |  |  |  |  |
| **Cost associated with diseases (Societal perspective)** |  |  |  |  |  |  |
|  |  |  |  |  |  |  |
| **CVD** | **4,746** | fixed |  |  |  | Liu, Maniadakis [31] |
| **Alzheimer** | **29,158** | fixed |  |  |  | Wimo, Reed [32] |
| **Dementia** | **23,190** | fixed |  |  |  | Sicras, Rejas [33] |
| **Falls** | **4,444** | fixed |  |  |  | Hartholt, van Beeck [34] |
| **Fractures** | **13,078** | fixed |  |  |  | Hartholt, van Beeck [34] |
| **Stroke** | **43,938** | fixed |  |  |  | Patel, Berdunov [24] |
| **Stroke (after 1st year)** | **29,203** | fixed |  |  |  | Patel, Berdunov [24] |
| **Diabetes** | **7,409** | fixed |  |  |  | Hex, Bartlett [35] |
|  |  |  |  |  |  |  |
| **Cost of the SITLESS intervention and control (cost/person)** |  |  |  |  |  |  |
|  |  |  |  |  |  |  |
| **SMS+ERS** | **286** | fixed |  |  |  | SITLESS RCT[22] |
| **ERS** | **186** | fixed |  |  |  | SITLESS RCT [22] |
| **UC** | **18** | fixed |  |  |  | SITLESS RCT [22] |
|  |  |  |  |  |  |  |
| **Utility values** |  |  |  |  |  |  |
| **Disease** |  |  | **r(α)** | β |  |  |
|  |  |  |  |  |  |  |
| **Alzheimer`s disease** | **0.60** | Gamma | 100.000 | 0.006 | 0.06 | Vandepitte, Putman [36] |
| **Dementia** | **0.75** | Gamma | 9.000 | 0.083 | 0.25 | Orgeta, Edwards [37] |
| **Falls** | **0.62** | Gamma | 7.267 | 0.085 | 0.23 | Bjerk, Brovold [38] |
| **Fractures (disutility)** | **0.14** |  |  |  |  | Karnon, Afzali [39] |
| **Stroke** | **0.62** | Gamma | 3.33 | 0.19 | 0.34 | Pickard, Johnson [40] |
| **CVD** | **0.72** | Gamma | 8.73 | 0.08 | 0.243 | Lacey and Walters [41] |
| **Diabetes** | **0.77** | Gamma | 8.133 | 0.095 | 0.27 | Clarke, Gray [42] |
| **Physical activity states** |  |  |  |  |  |  |
| **Inactive** | **0.760** |  | 0.736 | 0.784 | 0.012 | SITLESS RCT [22] |
| **Recommended activity** | **0.797** |  | 0.776 | 0.817 | 0.010 | SITLESS RCT [22] |
|  |  |  |  |  |  |  |
|  |  |  |  |  |  |  |
| **Mortality** |  |  |  |  |  |  |
| Inactive to death | 0.0150 | Fixed |  |  |  | Ekelund, Tarp [18] |
| Recommended activity to death | 0.0050 | Fixed |  |  |  | Ekelund, Tarp [18] |
| Background mortality | ***Age-specific, Life Tables*** | |  |  |  | ONS [21] |
| Alzheimer to death | 0.074 | Fixed |  |  |  | Garcia-Ptacek, Farahmand [43] |
| Dementia to death | 0.160 | Fixed |  |  |  | Garcia-Ptacek, Farahmand [43] |
| Falls to death | 0.010 | Fixed |  |  |  | Dunn, Rudberg [44] |
| Fractures to death | 0.235 | Fixed |  |  |  | Dunn, Rudberg [44] |
| Stroke to death | 0.4 | Fixed |  |  |  | Brønnum-Hansen, Davidsen [45] |
| CVD to death | 0.002 | Fixed |  |  |  | Wilmot, O’Flaherty [46] |
| Diabetes to death | 0.015 | Fixed |  |  |  | Hendriks, van Hateren [47] |

**Supplementary Appendix S3. The cost of the SITLESS intervention**

Tables 1, 2 and 3 show the breakdown of the SMS, ERS and UC costs, as well as the mean total cost and cost/participant of the SMS, ERS and UC intervention, respectively, for each country. The total cost and cost/participant calculated considering the opportunity cost (i.e. benefit foregone) in terms of venue, participants and staff travel cost is also shown. All costs have been reported in Euros, and adjusted using PPP for a base year.

**Table 1 Cost of the SMS intervention, by centre**

| **SMS INTERVENTION** | **TOTAL COST (PPP adjustment for values in £) Values in € 2017** | | | |
| --- | --- | --- | --- | --- |
| **COST CATEGORY** | **ODENSE** | **BARCELONA** | **BELFAST** | **ULM** |
| N | 113 | 118 | 108 | 116 |
| **Venue** |  |  |  |  |
| *Opportunity cost* | 3481.1 | 3726.1 | 2623.5 | 2437.7 |
| *Rent cost* | 0.0 | 0.0 | 0.0 | 180.0 |
| **Staff Cost** |  |  |  |  |
| *Individual session* | 3382.2 | 3154.7 | 3135.0 | 2455.0 |
| *Group sessions* | 2668.4 | 3317.6 | 1952.0 | 2153.9 |
| *Telephone calls* | 3828.3 | 3445.6 | 2508.0 | 4110.4 |
| **Travel costs** |  |  |  |  |
| Travel cost (staff) | 58.1 | 213.3 | 244.1 | 48.4 |
| Travel cost staff opportunity cost | 5237.2 | 1391.3 | 207.5 | 72.6 |
| Travel cost (participants) | 1877.3 | 0.0 | 6142.0 | 3980.0 |
| Travel cost (participants opportunity cost) | 2006.9 | 1544.6 | 580.9 | 368.3 |
| **Equipment** |  |  |  |  |
| Equipment | 2048.0 | 4249.8 | 1278.0 | 1656.5 |
| **Other costs** | 0.0 | 0.0 | 0.0 | 0.0 |
| **Total cost (I)** | **13862.3** | **14381.0** | **15259.0** | **14584.2** |
| **Cost/participant (I)** | **122.7** | **121.9** | **141.3** | **125.7** |
| **Total cost (II) (including travel and venue opportunity cost)** | **22652.0** | **20829.7** | **12284.8** | **13254.3** |
| **Cost/participant (II)** | **200.5** | **176.5** | **113.7** | **114.3** |

**Table 2 Cost of the ERS intervention, by centre**

| **ERS INTERVENTION** | **TOTAL COST (PPP adjustment for values in £) Values in € 2017** | | | |
| --- | --- | --- | --- | --- |
| **COST CATEGORY** | **ODENSE** | **BARCELONA** | **BELFAST** | **ULM** |
| N | 112 | 118 | 104 | 113 |
| **Venue** |  |  |  |  |
| *Opportunity cost* | 8320.0 | 6800.0 | 7192.6 | 5866.7 |
| *Rent cost* | 2164.5 | 0.0 | 0.0 | 1760.0 |
| **Staff Cost** |  |  |  |  |
| *Group sessions* | 14450.7 | 11810.6 | 12227.5 | 10189.6 |
| *Telephone calls* | 972.6 | 205.0 | 43.4 | 359.0 |
| **Travel costs** |  |  |  |  |
| Travel cost (staff) | 1664.0 | 850.0 | 1764.9 | 352.0 |
| Travel cost staff opportunity cost | 597.3 | 1574.8 | 2678.9 | 4075.8 |
| Travel cost (participants) | 7168.0 | 0.0 | 9251.2 | 7232.0 |
| Travel cost (participants opportunity cost) | 10035.2 | 7723.9 | 969.0 | 10570.8 |
| **Equipment** |  |  |  |  |
| Equipment | 188.8 | 199.0 | 175.3 | 190.5 |
| Other costs |  |  |  |  |
| **Other costs** | 208.4 | 191.1 |  | 17.4 |
| **Total cost (I)** | **26817.1** | **13255.6** | **23462.4** | **20100.4** |
| **Cost/participant (I)** | **239.4** | **112.3** | **225.6** | **177.9** |
| **Total cost (II) (including travel and venue opportunity cost)** | **34773.1** | **28504.3** | **23286.9** | **31269.7** |
| **Cost/participant (II)** | **310.5** | **241.6** | **223.9** | **276.7** |

**Notes**: travel cost of participants in Barcelona are zero since all participants walked to the exercise facility (the primary health care centre located in their neighbourhood). The category ‘other cost’ includes the preparation time for trainers.

**Table 3 Cost of the UC intervention, by centre**

| **UC INTERVENTION** | **TOTAL COST (PPP adjustment for values in £) Values in € 2017** | | | |
| --- | --- | --- | --- | --- |
| **COST CATEGORY** | **ODENSE** | **BARCELONA** | **BELFAST** | **ULM** |
| N | 113 | 120 | 109 | 116 |
| **Venue** |  |  |  |  |
| *Opportunity cost* | 240.0 | 440.0 | 408.7 | 360.0 |
| *Rent cost* | 26.6 | 0.0 | 0.0 | 0.0 |
| **Staff Cost** |  |  |  |  |
| *Group sessions* | 625.3 | 764.2 | 671.6 | 937.9 |
| *Telephone calls* | 260.5 | 416.9 | 13.9 | 335.8 |
| **Travel costs** |  |  |  |  |
| Travel cost (staff) | 0.0 | 55.0 | 84.7 | 0.0 |
| Travel cost staff opportunity cost | 124.7 | 31.6 | 128.5 | 0.0 |
| Travel cost (participants) | 485.9 | 0.0 | 1180.1 | 464.0 |
| Travel cost (participants opportunity cost) | 632.8 | 613.0 | 309.0 | 339.1 |
| **Equipment** |  |  |  |  |
| Equipment | 0.0 | 0.0 | 0.0 | 5.0 |
| Other costs |  |  |  |  |
| **Other costs** | 480.0 | 0.0 | 224.8 | 270.0 |
| **Total cost (1) excluding opportunity cost** | **1878.3** | **1236.1** | **2175.0** | **2012.7** |
| **Cost/participant (1)** | **16.6** | **10.3** | **20.0** | **17.4** |
| **Total cost (2) including travel and venue opportunity cost** | **2363.3** | **2265.7** | **1756.5** | **2247.8** |
| **Cost/participant (2)** | **20.9** | **18.9** | **16.1** | **19.4** |

**Supplementary Appendix S4: WTP Thresholds**

| **Country** | **WTP value €** | **WTP value £** |
| --- | --- | --- |
| **UK** | **20,433** | **20,000** |
| **UK** | **30,650** | **30,000** |
| **Spain** | **27,300** |  |
| **Denmark** | **37,700** |  |

**Supplementary Appendix S5 Within-Trial sensitivity analysis**

**Table 1: Sensitivity analysis considering alternative definitions of costs**

|  | **Arm** | |  |  |
| --- | --- | --- | --- | --- |
|  | **ERS+SMS** | **ERS** | **Difference** | **ICER**  (Incremental Cost/Incremental QALY) |
| QALY | 1.449 | 1.4323 |  |  |
| Societal cost | 12543 | 12266 | **277** | **10375** |
| Cost falls+social cost | 2662 | 1812 | **850** | **31835** |
| Cost of intervention +20% | 3205 | 3045 | **160** | **5993** |
| Cost of intervention -20% | 3138 | 3069 | **69** | **2584** |
| Opportunity cost of intervention | 3295 | 3137 | **158** | **5918** |
|  |  |  |  |  |
|  | **Arm** | |  |  |
|  | **ERS+SMS** | **UC** | **Difference** |  |
| QALY | 1.449 | 1.4357 |  |  |
| Societal cost | 12543 | 12380 | **163** | **12256** |
| Cost falls+social cost | 2662 | 454 | **2208** | **166015** |
| Cost of intervention +20% | 3205 | 2662 | **543** | **40827** |
| Cost of intervention -20% | 3138 | 2683 | **455** | **34211** |
| Opportunity cost of intervention | 3295 | 2603 | **692** | **52030** |

**Table 2: Sensitivity analysis considering the one country approach to evaluate costs and outcomes**

| **Results table: within trial cost, QALY, Capability QALY and ICER One-country approach** | | | | |
| --- | --- | --- | --- | --- |
|  | **Arm** |  |  |  |
|  | **ERS+SMS** | **ERS** | **Difference** | **ICER (**€) |
| Cost | 4802 | 4859 | **-57** |  |
| QALY | 1.3737 | 1.3429 | **0.0308** | **SMS+ERS dominates** |
| YFC | 1.498 | 1.4826 | **0.0154** | **SMS+ERS dominates** |
|  |  |  |  |  |
|  | **ERS+SMS** | **UC** | **Difference** |  |
| Cost | 4802 | 4339 | **463** |  |
| QALY | 1.3737 | 1.3616 | **0.0121** | **38264** |
| YFC | 1.498 | 1.4921 | **0.0059** | **78475** |

**Table 3: Sensitivity analysis considering departure from the MAR assumption: MNAR scenarios**

| **Results table: MNAR scenarios** | | | | | |
| --- | --- | --- | --- | --- | --- |
|  |  | **ICER QALY (€)** |  | **ICER CQALY (€)** |  |
| **Scenario** |  | **SMS+ERS VS ERS** | **SMS+ERS VS UC** | **SMS+ERS VS ERS** | **SMS+ERS VS UC** |
| **0** | **baseline** | **3535** | **ICER>£30K** | **9973** | **ICER>£30K** |
| **1** | **Missing costs and qaly/cap qaly are 10% lower in all arms** | **3764** | **18446** | **10195** | **ICER>£30K** |
| **2** | **Missing costs and qaly/cap qaly are 10% higher in all arms** | **3335** | **ICER>£30K** | **9641** | **SMS+ERS dominated** |
| **3** | **Missing costs and qaly/cap qaly are 10% higher in the SMS arm** | **2797** | **10556** | **3745** | **13432** |
| **4** | **Missing costs and qaly/cap qaly are 10% lower in the SMS arm** | **SMS+ERS dominated** | **SMS+ERS dominated** | **SMS+ERS dominated** | **SMS+ERS dominated** |
| **5** | **Missing qaly/cap qaly are 10% lower in all arms** | **3889** | **17639** | **10531** | **ICER>£30K** |
| **6** | **Missing qaly/cap qaly are 10% higher in all arms** | **3224** | **ICER>£30K** | **9319** | **SMS+ERS dominated** |
| **7** | **Missing qaly/cap qaly are 10% higher in the SMS arm** | **1530** | **8860** | **2049** | **11275** |
| **8** | **Missing qaly/cap qaly are 10% lower in the SMS arm** | **SMS+ERS dominated** | **SMS+ERS dominated** | **SMS+ERS dominated** | **SMS+ERS dominated** |

**Supplementary Appendix S6 Long Term results- Scenario analyses**

The following three scenario analyses have been conducted to assess the robustness of results to key assumptions, specifically:

- **Decay of intervention effect (Scenario I)**. Testing the robustness of results to the assumptions which have been made in relation to the persistence of the intervention effect is crucial, in consideration of the potential of the behavioural SMS component to mitigate the lack of long-term commitment which is often associated with PA interventions. For this reason, in Scenario I the transition rate between PA states has been calculated as the rate of transition into a different PA state between baseline and 22 months. This is different from the base-case scenario, where, as explained in Table 3, transitions rates were calculated in the first year considering movements between baseline and post-intervention, while subsequent transitions consider movements between post-intervention and 22 months. In other terms, instead of modelling the decay of the PA levels considering a different transition rate from year 2 on, in Scenario I we consider the average rate, calculated over the entire time span.
- **Comorbidity (Scenario II)** The SITLESS model does not account explicitly for comorbidities, since once in a disease state, participants can only stay in that state or move to death. Therefore, in Scenario II, a utility decrement has been applied to account for the lower utility associated to comorbidities [48]. In this way, besides indirectly accounting for the effect of comorbidities considering age-specific mortality rates, we further consider the reduction in utility associated to the coexistence of two or more diseases. A disutility value of 3.88% (95%CI: −5.37%,-2.39) has been applied; this has been multiplied by the average number of disease of a SITLESS participants
- **Sedentary behaviour (Scenario III)** In Scenario III, a mortality decrement was applied to account for the detrimental effect of SB, i.e. the increase in mortality risk caused by an excessive SB, which may arise regardless of meeting the recommended PA levels [18]. The mortality risk for active and inactive individuals has been adjusted using the hazard ratio of 1.27 estimated by Ekelund et al [18]’.

Table 1 below shows the cost-effectiveness results for each of these scenarios, considering the 5 and 15 years time horizon.

**Table 1: Incremental costs, incremental QALY and ICER for the long-term model scenario analyses**

|  | **Arm** | Total costs | Total QALYs | Comparison | Incremental costs (CI) | Incremental QALY (CI) | **ICER** |
| --- | --- | --- | --- | --- | --- | --- | --- |
| 5 YEARS Time horizon | **SMS+ERS** | 13,290 | 2.6591 |  |  |  |  |
| **Scenario I** | **ERS** | 13,250 | 2.6638 | SMS+ERS vs. ERS | 40(-57; 151) | -0.0047 (-0.0162; 0.0050) | ***SMS+ERS is dominated*** |
| ***Transition rates calculated over 22 months*** | **UC** | 13,312 | 2.6566 | SMS+ERS vs. UC | -22 (-60:135) | 0.0025 (-.00077; 0.0139) | ***SMS+ERS dominates*** |
|  |  |  |  |  |  |  |  |
| 15 YEARS Time horizon | **SMS+ERS** | 45,588 | 6.9644 | SMS+ERS vs. ERS |  |  |  |
| **Scenario I** | **ERS** | 45,531 | 6.9763 | SMS+ERS vs. UC | 57 (-178; 325) | -0.0119 (-0.0471; 0.0217) | ***SMS+ERS is dominated*** |
| ***Transition rates calculated over 22 months*** | **UC** | 45,583 | 6.9634 | SMS+ERS vs. UC | 5 (-286; 250) | 0.0010 (-0.0356; 0.0419) | ***5075*** |
|  |  |  |  |  |  |  |  |
| 5 YEARS Time horizon | **SMS+ERS** | 13,294 | 2.4032 |  |  |  |  |
| **Scenario II** | **ERS** | 13,326 | 2.4012 | SMS+ERS vs. ERS | -32 (-140; 57) | 0.0020 (-0.0036; 0.0102) | ***SMS+ERS dominates*** |
| ***Comorbidities utility decrement*** | **UC** | 13,347 | 2.4000 | SMS+ERS vs. UC | -52 (-194; 51) | 0.0032 (-0.0032; 0.0136) | ***SMS+ERS dominates*** |
|  |  |  |  |  |  |  |  |
| 15 YEARS Time horizon | **SMS+ERS** | 45,634 | 6.5276 |  |  |  |  |
| **Scenario II** | **ERS** | 45,604 | 6.5285 | SMS+ERS vs. ERS | 30 (-173; 283) | -0.0008 (-0.0279; 0.0239) | ***SMS+ERS is dominated*** |
| ***Comorbidities utility decrement*** | **UC** | 45,628 | 6.5252 | SMS+ERS vs. UC | 6 (-243; 260) | 0.0024 (-0.0233; 0.0284) | ***2611*** |

Note: credibility intervals in parenthesis

**Supplementary Appendix S7 Cost effectiveness planes and CEACs**


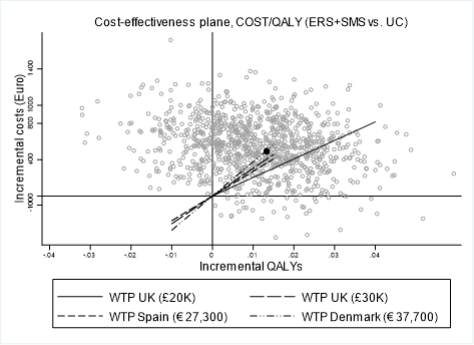


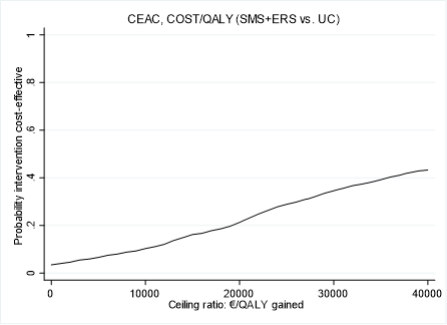


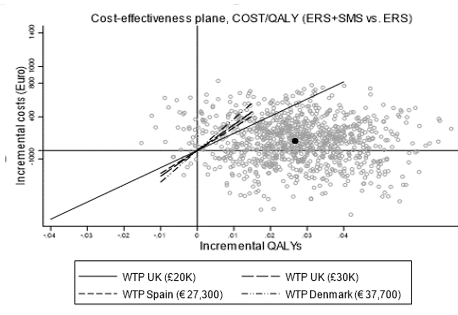

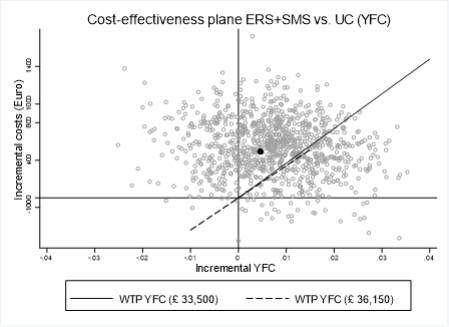


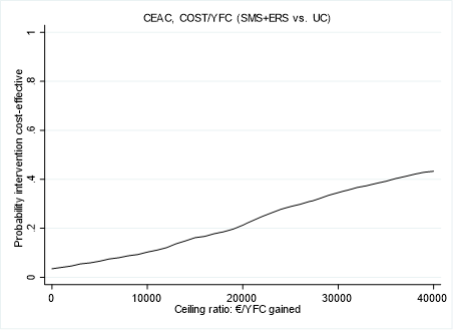


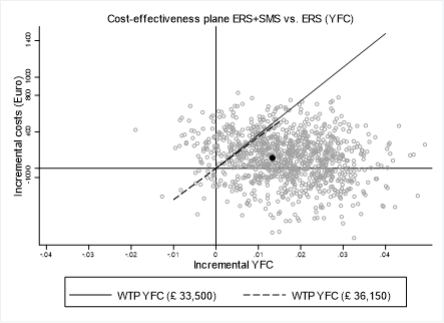


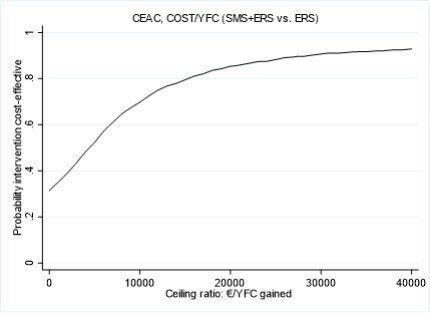


**Supplementary Appendix S8: summary statistics**

**SITLESS primary outcomes**

| **Axis1 CPM** |  |  |  |
| --- | --- | --- | --- |
|  | **Mean** | **95% CI** | |
| ERS+SMS | 197.15 | 186.21 | 208.09 |
| ERS | 198.45 | 188.85 | 208.06 |
| UC | 192.41 | 183 | 201.81 |
| **% Sedentary Time** |  |  |  |
|  | **Mean** | **95% CI** | |
| ERS+SMS | 78.81% | 78.11% | 79.52% |
| ERS | 78.54% | 77.88% | 79.21% |
| UC | 79.11 | 78.49% | 79.74% |

**Baseline variables**

|  | **Mean** | **Standard deviation** | **Minimum** | **Maximum** |
| --- | --- | --- | --- | --- |
| **Age at randomisation into SITLESS** |  |  |  |  |
| ERS+SMS | 75.14 | 6.16 | 64 | 92 |
| ERS | 75.19 | 6.28 | 65 | 91 |
| UC | 75.48 | 6.40 | 64 | 93 |
|  |  |  |  |  |
| **Number of comorbidities** | |  |  |  |
|  |  |  |  |  |
| Control | 2.94 | 2.18 | 0 | 10 |
| ERS | 2.83 | 1.97 | 0 | 10 |
| SMS+ERS | 3.01 | 2.04 | 0 | 16 |

| **Gender** | **Male** | **Female** |  |  |  |
| --- | --- | --- | --- | --- | --- |
| ERS+SMS | 38.20% | 61.80% |  |  |  |
| ERS | 38.30% | 61.70% |  |  |  |
| UC | 38.20% | 61.80% |  |  |  |
| **Marital status** | **Single** | **Married/Stable relation** | **Widow/Widower** | **Divorced** |  |
| ERS+SMS | 9.09% | 57.73% | 25.91% | 12.27% |  |
| ERS | 8.94% | 52.98% | 26.83% | 11.24% |  |
| UC | 8.80% | 52.55% | 28.47% | 10.19% |  |
| **Living arrangements** | **Living alone** | **Not living alone** |  |  |  |
| ERS+SMS | 41.32% | 58.68% |  |  |  |
| ERS | 38.03% | 61.97% |  |  |  |
| UC | 39.96% | 60.04% |  |  |  |
| **Education** | **I do not know how to read and write** | **I know how to read an write** | **Primary education** | **Secondary education** | **University** |
| ERS+SMS | 0.45% | 3.34% | 20.27% | 52.78% | 23.16% |
| ERS | 0.46% | 2.97% | 21.46% | 51.83% | 23.29% |
| UC | 0.22% | 1.79% | 20.98% | 55.36% | 21.65% |

**References**

1. Sculpher, M., F. Pang, A. Manca, M. Drummond, S. Golder, H. Urdahl, L. Davies, and A. Eastwood, *Generalisability in economic evaluation studies in healthcare: a review and case studies.* 2004.

2. Briggs, A., H. Glick, G. Lozano-Ortega, M. Spencer, P. Calverley, P. Jones, and J. Vestbo, *Is treatment with ICS and LABA cost-effective for COPD? Multinational economic analysis of the TORCH study.* European Respiratory Journal, 2010. **35**(3): p. 532-539.

3. Drummond, M., M. Barbieri, J. Cook, H.A. Glick, J. Lis, F. Malik, S.D. Reed, F. Rutten, M. Sculpher, and J. Severens, *Transferability of economic evaluations across jurisdictions: ISPOR Good Research Practices Task Force report.* Value in health, 2009. **12**(4): p. 409-418.

4. Manca, A., N. Hawkins, and M.J. Sculpher, *Estimating mean QALYs in trial‐based cost‐effectiveness analysis: the importance of controlling for baseline utility.* Health economics, 2005. **14**(5): p. 487-496.

5. Faria, R., M. Gomes, D. Epstein, and I.R. White, *A guide to handling missing data in cost-effectiveness analysis conducted within randomised controlled trials.* Pharmacoeconomics, 2014. **32**(12): p. 1157-1170.

6. White, I.R., N.J. Horton, J. Carpenter, and S.J. Pocock, *Strategy for intention to treat analysis in randomised trials with missing outcome data.* Bmj, 2011. **342**: p. d40.

7. White, I.R., P. Royston, and A.M. Wood, *Multiple imputation using chained equations: issues and guidance for practice.* Statistics in medicine, 2011. **30**(4): p. 377-399.

8. Schomaker, M. and C. Heumann, *Bootstrap inference when using multiple imputation.* Statistics in medicine, 2018. **37**(14): p. 2252-2266.

9. Leurent, B., M. Gomes, R. Faria, S. Morris, R. Grieve, and J.R. Carpenter, *Sensitivity analysis for not-at-random missing data in trial-based cost-effectiveness analysis: a tutorial.* PharmacoEconomics, 2018. **36**(8): p. 889-901.

10. Roux, L., M. Pratt, T.O. Tengs, M.M. Yore, T.L. Yanagawa, J. Van Den Bos, C. Rutt, R.C. Brownson, K.E. Powell, and G. Heath, *Cost effectiveness of community-based physical activity interventions.* American journal of preventive medicine, 2008. **35**(6): p. 578-588.

11. Frew, E.J., M. Bhatti, K. Win, A. Sitch, A. Lyon, M. Pallan, and P. Adab, *Cost-effectiveness of a community-based physical activity programme for adults (Be Active) in the UK: an economic analysis within a natural experiment.* Br J Sports Med, 2014. **48**(3): p. 207-212.

12. UK Chief Medical Officers, *UK Chief Medical Officers' Physical Activity Guidelines.* 2019.

13. NICE. *Methods for the development of NICE public health guidance. 2012*

14. Jefferis, B.J., T.J. Parsons, C. Sartini, S. Ash, L.T. Lennon, O. Papacosta, R.W. Morris, S.G. Wannamethee, I.-M. Lee, and P.H. Whincup, *Does total volume of physical activity matter more than pattern for onset of CVD? A prospective cohort study of older British men.* International journal of cardiology, 2019. **278**: p. 267-272.

15. Ainsworth, B.E., W.L. Haskell, M.C. Whitt, M.L. Irwin, A.M. Swartz, S.J. Strath, W.L. O Brien, D.R. Bassett, K.H. Schmitz, and P.O. Emplaincourt, *Compendium of physical activities: an update of activity codes and MET intensities.* Medicine and science in sports and exercise, 2000. **32**(9; SUPP/1): p. S498-S504.

16. Abbott, R.D., L.R. White, G.W. Ross, K.H. Masaki, J.D. Curb, and H. Petrovitch, *Walking and dementia in physically capable elderly men.* Jama, 2004. **292**(12): p. 1447-1453.

17. Tomata, Y., S. Zhang, Y. Sugawara, and I. Tsuji, *Impact of time spent walking on incident dementia in elderly Japanese.* International Journal of Geriatric Psychiatry, 2019. **34**(1): p. 204-209.

18. Ekelund, U., J. Tarp, J. Steene-Johannessen, B.H. Hansen, B. Jefferis, M.W. Fagerland, P. Whincup, K.M. Diaz, S.P. Hooker, and A. Chernofsky, *Dose-response associations between accelerometry measured physical activity and sedentary time and all cause mortality: systematic review and harmonised meta-analysis.* bmj, 2019. **366**: p. l4570.

19. Kolovos, S., A.P. Finch, H.P. Van Der Ploeg, F. Van Nassau, H.M. Broulikova, A. Baka, S. Treweek, C.M. Gray, J.G. Jelsma, and C. Bunn, *Five-year cost-effectiveness analysis of the European Fans in Training (EuroFIT) physical activity intervention for men versus no intervention.* International Journal of Behavioral Nutrition and Physical Activity, 2020. **17**(1): p. 1-13.

20. Dyer, M.T., K.A. Goldsmith, L.S. Sharples, and M.J. Buxton, *A review of health utilities using the EQ-5D in studies of cardiovascular disease.* Health and quality of life outcomes, 2010. **8**(1): p. 1-12.

21. ONS, *National life tables: UK*.

22. SITLESS, *Exercise Referral Schemes enhanced by Self-Management Strategies to battle sedentary behaviour (unpublished report).* European Commission,, 2020.

23. Lacey, B., J. Golledge, B.B. Yeap, S. Lewington, P.E. Norman, L. Flicker, O.P. Almeida, and G.J. Hankey, *Physical activity and vascular disease in a prospective cohort study of older men: The Health In Men Study (HIMS).* BMC geriatrics, 2015. **15**(1): p. 164.

24. Patel, A., V. Berdunov, Z. Quayyum, D. King, M. Knapp, and R. Wittenberg, *Estimated societal costs of stroke in the UK based on a discrete event simulation.* Age and ageing, 2020. **49**(2): p. 270-276.

25. Podewils, L.J., E. Guallar, L.H. Kuller, L.P. Fried, O.L. Lopez, M. Carlson, and C.G. Lyketsos, *Physical activity, APOE genotype, and dementia risk: findings from the Cardiovascular Health Cognition Study.* American journal of epidemiology, 2005. **161**(7): p. 639-651.

26. Larsson, S.C. and A. Wolk, *The role of lifestyle factors and sleep duration for late-onset dementia: A cohort study.* Journal of Alzheimer's Disease, 2018. **66**(2): p. 579-586.

27. Laurin, D., R. Verreault, J. Lindsay, K. MacPherson, and K. Rockwood, *Physical activity and risk of cognitive impairment and dementia in elderly persons.* Archives of neurology, 2001. **58**(3): p. 498-504.

28. Buchner, D.M., E. Rillamas‐Sun, C. Di, M.J. LaMonte, S.W. Marshall, J. Hunt, Y. Zhang, D.E. Rosenberg, I.M. Lee, and K.R. Evenson, *Accelerometer‐measured moderate to vigorous physical activity and incidence rates of falls in older women.* Journal of the American Geriatrics Society, 2017. **65**(11): p. 2480-2487.

29. Jefferis, B.J., P.H. Whincup, O. Papacosta, and S.G. Wannamethee, *Protective effect of time spent walking on risk of stroke in older men.* Stroke, 2014. **45**(1): p. 194-199.

30. InterAct Consortium, *Physical activity reduces the risk of incident type 2 diabetes in general and in abdominally lean and obese men and women: the EPIC–InterAct Study.* Diabetologia, 2012. **55**(7): p. 1944-1952.

31. Liu, J., N. Maniadakis, A. Gray, and M. Rayner, *The economic burden of coronary heart disease in the UK.* Heart, 2002. **88**(6): p. 597-603.

32. Wimo, A., C.C. Reed, R. Dodel, M. Belger, R.W. Jones, M. Happich, J.M. Argimon, G. Bruno, D. Novick, and B. Vellas, *The GERAS study: a prospective observational study of costs and resource use in community dwellers with Alzheimer's disease in three European countries–study design and baseline findings.* Journal of Alzheimer's Disease, 2013. **36**(2): p. 385-399.

33. Sicras, A., J. Rejas, S. Arco, E. Flores, G. Ortega, A. Esparcia, A. Suárez, and M. Gordillo, *Prevalence, resource utilization and costs of vascular dementia compared to Alzheimer’s dementia in a population setting.* Dementia and geriatric cognitive disorders, 2005. **19**(5-6): p. 305-315.

34. Hartholt, K.A., E.F. van Beeck, S. Polinder, N. van der Velde, E.M. van Lieshout, M.J. Panneman, T.J. van der Cammen, and P. Patka, *Societal consequences of falls in the older population: injuries, healthcare costs, and long-term reduced quality of life.* Journal of Trauma and Acute Care Surgery, 2011. **71**(3): p. 748-753.

35. Hex, N., C. Bartlett, D. Wright, M. Taylor, and D. Varley, *Estimating the current and future costs of Type 1 and Type 2 diabetes in the UK, including direct health costs and indirect societal and productivity costs.* Diabetic medicine, 2012. **29**(7): p. 855-862.

36. Vandepitte, S., K. Putman, N. Van Den Noortgate, N. Verhaeghe, and L. Annemans, *Cost‐effectiveness of an in‐home respite care program to support informal caregivers of persons with dementia: A model‐based analysis.* International Journal of Geriatric Psychiatry, 2020. **35**(6): p. 601-609.

37. Orgeta, V., R.T. Edwards, B. Hounsome, M. Orrell, and B. Woods, *The use of the EQ-5D as a measure of health-related quality of life in people with dementia and their carers.* Quality of Life Research, 2015. **24**(2): p. 315-324.

38. Bjerk, M., T. Brovold, J.C. Davis, and A. Bergland, *Evaluating a falls prevention intervention in older home care recipients: a comparison of SF-6D and EQ-5D.* Quality of life research, 2019. **28**(12): p. 3187-3195.

39. Karnon, J., H.H.A. Afzali, G.V.A.A. Putro, P.W. Thant, A. Dompok, I. Cox, O.H. Chikhwaza, X. Wang, M.M. Mwangangi, and M. Farransahat, *A Cost-Effectiveness model for frail older persons: Development and application to a physiotherapy-based intervention.* Applied Health Economics and Health Policy, 2017. **15**(5): p. 635-645.

40. Pickard, A.S., J.A. Johnson, D.H. Feeny, A. Shuaib, K. Carriere, and A.M. Nasser, *Agreement between patient and proxy assessments of health-related quality of life after stroke using the EQ-5D and Health Utilities Index.* Stroke, 2004. **35**(2): p. 607-612.

41. Lacey, E. and S. Walters, *Continuing inequality: gender and social class influences on self perceived health after a heart attack.* Journal of Epidemiology & Community Health, 2003. **57**(8): p. 622-627.

42. Clarke, P., A. Gray, and R. Holman, *Estimating utility values for health states of type 2 diabetic patients using the EQ-5D (UKPDS 62).* Medical Decision Making, 2002. **22**(4): p. 340-349.

43. Garcia-Ptacek, S., B. Farahmand, I. Kåreholt, D. Religa, M.L. Cuadrado, and M. Eriksdotter, *Mortality risk after dementia diagnosis by dementia type and underlying factors: a cohort of 15,209 patients based on the Swedish Dementia Registry.* Journal of Alzheimer's Disease, 2014. **41**(2): p. 467-477.

44. Dunn, J.E., M.A. Rudberg, S.E. Furner, and C.K. Cassel, *Mortality, disability, and falls in older persons: the role of underlying disease and disability.* American Journal of Public Health, 1992. **82**(3): p. 395-400.

45. Brønnum-Hansen, H., M. Davidsen, and P. Thorvaldsen, *Long-term survival and causes of death after stroke.* Stroke, 2001. **32**(9): p. 2131-2136.

46. Wilmot, K.A., M. O’Flaherty, S. Capewell, E.S. Ford, and V. Vaccarino, *Coronary heart disease mortality declines in the United States from 1979 through 2011: evidence for stagnation in young adults, especially women.* Circulation, 2015. **132**(11): p. 997-1002.

47. Hendriks, S.H., K.J. van Hateren, K.H. Groenier, G.W. Landman, A.H. Maas, H.J. Bilo, and N. Kleefstra, *Sex differences in survival of patients with type 2 diabetes in primary care (ZODIAC-50).* BMJ open, 2017. **7**(10): p. e015870.

48. Makovski, T.T., S. Schmitz, M.P. Zeegers, S. Stranges, and M. van den Akker, *Multimorbidity and quality of life: systematic literature review and meta-analysis.* Ageing research reviews, 2019. **53**: p. 100903.
